# Sex Pheromone of the Alfalfa Plant Bug, *Adelphocoris lineolatus*: Pheromone Composition and Antagonistic Effect of 1-Hexanol (Hemiptera: Miridae)

**DOI:** 10.1007/s10886-021-01273-y

**Published:** 2021-04-19

**Authors:** Sándor Koczor, József Vuts, John C. Caulfield, David M. Withall, André Sarria, John A. Pickett, Michael A. Birkett, Éva Bálintné Csonka, Miklós Tóth

**Affiliations:** 1 Plant Protection Institute, Centre for Agricultural Research, Eötvös Loránd Research Network (ELKH), H-1022 Herman Ottó u. 15, Budapest, Hungary; 2 Department of Biointeractions and Crop Protection, Rothamsted Research, Harpenden, Hertfordshire AL5 2JQ UK; 3 Present Address: Biobab R&D, S. L. Calle Patones, s/n. Parcela 28.3 PI Ventorro del Cano, Alcorcón, 28925 Madrid, Spain; 4 Present Address: School of Chemistry, Cardiff University, Cardiff, Wales CF10 3AT UK

**Keywords:** *Adelphocoris lineolatus*, Sex pheromone, Field attraction, Electroantennography, Miridae

## Abstract

The sex pheromone composition of alfalfa plant bugs, *Adelphocoris lineolatus* (Goeze), from Central Europe was investigated to test the hypothesis that insect species across a wide geographical area can vary in pheromone composition. Potential interactions between the pheromone and a known attractant, (*E*)-cinnamaldehyde, were also assessed. Coupled gas chromatography-electroantennography (GC-EAG) using male antennae and volatile extracts collected from females, previously shown to attract males in field experiments, revealed the presence of three physiologically active compounds. These were identified by coupled GC/mass spectrometry (GC/MS) and peak enhancement as hexyl butyrate, (*E*)-2-hexenyl butyrate and (*E*)-4-oxo-2-hexenal. A ternary blend of these compounds in a 5.4:9.0:1.0 ratio attracted male *A. lineolatus* in field trials in Hungary. Omission of either (*E*)-2-hexenyl-butyrate or (*E*)-4-oxo-2-hexenal from the ternary blend or substitution of (*E*)-4-oxo-2-hexenal by (*E*)-2-hexenal resulted in loss of activity. These results indicate that this Central European population is similar in pheromone composition to that previously reported for an East Asian population. Interestingly, another EAG-active compound, 1-hexanol, was also present in female extract. When 1-hexanol was tested in combination with the ternary pheromone blend, male catches were reduced. This compound showed a dose-response effect with small doses showing a strong behavioral effect, suggesting that 1-hexanol may act as a sex pheromone antagonist in *A. lineolatus*. Furthermore, when (*E*)-cinnamaldehyde was field tested in combination with the sex pheromone, there was no increase in male catch, but the combination attracted both males and females. Prospects for practical application are discussed.

**Supplementary Information:**

The online version contains supplementary material available at 10.1007/s10886-021-01273-y.

## Introduction

Plant bugs (Heteroptera: Miridae) represent the most species-rich family of true bugs. Several species are pests, and some have an extremely wide spectrum of hosts (e.g., Holopainen and Varis 1991). Due to new pest control technologies and recent changes in regulation, there has been a marked and continuous decrease in the use of broad-spectrum insecticides in agriculture. As a consequence, pests considered previously to be of minor importance can become more important, as observed for genetically engineered lepidopteran-resistant crops, such as Bt-cotton (Lu et al. 2010). Furthermore, this effect may reach beyond the intended crop. Lu et al. (2010) found that broad-spectrum insecticide sprayings may result in ‘sink’ populations of a particular pest that, without such treatments, can reach high abundance and create ‘source’ populations, resulting in higher levels of damage in other crops. *Adelphocoris* species are among those pests that have gained increasing economic importance with the decrease in broad-spectrum insecticide use (Lu et al. 2008).

The alfalfa plant bug, *Adelphocoris lineolatus* (Goeze), occurs widely in the Palearctic, where it is a major pest of alfalfa, *Medicago sativa* L., Fabaceae (Benedek et al. 1970); several other potential hosts have also been reported (Golledge 1944; Peterson et al. 1992). Currently, the most serious economic impact of *Adelphocoris* spp., including *A. lineolatus,* is the damage caused to Bt-cotton in China (Lu et al. 2008; Wu et al. 2002).

Partially due to their increased economic importance, several reports on the chemical ecology of *Adelphocoris* species have been published recently, including pheromone identification of major pests of Bt-cotton in China, such as *A. fasciaticollis* Reuter (Zhang et al. 2015b), *A. suturalis* (Jakovlev) (Zhang et al. 2016) and *A. lineolatus* (Zhang et al. 2015a). The aim of such work is to develop species-specific detection and monitoring traps to aid pest management. Among these species, *A. lineolatus* has the widest distribution in the Palearctic, and has also been introduced to the Nearctic. Zhang et al. (2015a) identified hexyl butyrate, (*E*)-2-hexenyl butyrate and (*E*)-4-oxo-2-hexenal as components of the female sex pheromone of an east Asian population. As found in other pest insects with a wide distribution, pheromone composition can vary throughout a pest’s range. One example of this is *Agrotis segetum* (Denis and Schiffermüller), in which sex pheromone composition of populations in different geographic regions consist of different combinations of components (Tóth et al. 1992). Therefore, we wished to analyze a central European population of *A. lineolatus* to determine whether its sex pheromone differed from that of the east Asian population.

In addition, we wanted to test the behavioral effects of other chemicals on a central European population. (*E*)-4-Oxo-2-hexenal is a common component of plant bug pheromones that can be degraded by environmental conditions, including heat, light and oxidation. In previous studies on the chemical ecology of Miridae, special caution has been taken with its use. For instance, in one study, the compound was applied in separate bait dispensers and replaced on a daily basis to maintain activity (Byers et al. 2013). Yasuda and Higuchi (2012) reported that the level of (*E*)-4-oxo-2-hexenal decreased quickly in dispensers but that an increased dose attracted more male *Stenotus rubrovittatus* (Matsumura), another pestiferous plant bug species. Thus, in our study, we decided to test the compound in two dosages. We also tested (*E*)-2-hexenal, a more stable compound than (*E*)-4-oxo-2-hexenal, for possible analogous activity. Plant volatiles can affect sex pheromone production and activity in some insect species (Landolt and Phillips 1997). For example, in a closely related plant bug, *Lygus rugulipennis* (Poppius), host plant odors elicited increased sex pheromone production in females (Frati et al. 2009). Therefore, we wished to assess potential interactions between the sex pheromone and (*E*)-cinnamaldehyde, a floral volatile that attracts *A. lineolatus* (Koczor et al. 2012).

Overall, the major aims of this study were: 1) determine the pheromone composition of *A. lineolatus* in a population from central Europe, 2) test responses to two dosages of (*E*)-4-oxo-2-hexenal, 3) test responses to (*E*)-2-hexenal for potential analogous activity, and 4) assess responses to combinations of sex pheromone and (*E*)-cinnamaldehyde.

## Methods and Materials

### Insects for Experiments

Virgin *A. lineolatus* males and females were reared in the laboratory at 18:6 light:dark photoperiod, 26 °C and ca. 40% RH. Nymphs were collected by sweep-netting at alfalfa fields in Halásztelek, Pusztazámor and Tököl (Hungary), and taken to the laboratory, where they were reared on green bean pods in 12.5 × 17.5 cm glass jars covered with fine mesh. Freshly molted adults were removed from the rearing containers, identified, sexed and kept separate to ensure they were virgin when used. Adult bugs were kept in the same conditions as nymphs.

### Volatile Collection from Live Females

As field cage experiments with live bugs indicated the presence of a female-produced sex pheromone, headspace collections were performed with single *A. lineolatus* females on green bean pods, and with green bean pods alone as a control, for 1 d (20–24 h) or 3 d (71–72 h). For preparation of headspace collections, two methods were used. The bugs and green bean pods were placed in 200 ml glass containers of a closed-loop stripping apparatus (CLSA, Boland et al. 1984), equipped with a DC12/16NK vacuum pump (Erich Fürgut GmbH, Tannheim, Germany) with an airflow of ca. 5.0 l.min^−1^ and a collection filter containing 5 mg activated charcoal (Brechbühler AG, Schlieren, Switzerland). Trapped volatiles were eluted from the charcoal filter with 25 μl dichloromethane (Merck KGaA, Darmstadt, Germany).

To determine pheromone emission, dynamic headspace collection (air entrainment) (Birkett 2010) was carried out with single *A. lineolatus* females on green bean pods for 24 h under a 14:10 L:D photoperiod, 20 °C and ca. 50% RH. The material to be sampled was placed in a 380 ml glass jar, and activated charcoal-filtered (Capillary-Grade Hydrocarbon Trap; Thames Restek Ltd., High Wycombe, UK) air pumped (Pye volatile collection kits, Kings Walden, UK) through the inlet port at 600 ml.min^−1^. Air subsequently passed over the material in the jar and headspace volatiles were adsorbed on Porapak Q filters (0.05 g, 50/80 mesh; Supelco) on the outlet port, through which air was drawn at 500 ml.min^−1^. All connections in the air entrainment setup used PTFE tubing. Prior to entrainment, Porapak Q filters were washed with diethyl ether and conditioned at 132 °C for 2 h with an activated charcoal-filtered nitrogen stream. Entrained volatiles were eluted with 750 μl redistilled diethyl ether and stored in 1.1 ml glass microvials at −20 °C until analysis. Glass jars were washed with detergent (Teepol), acetone and distilled water, and baked overnight at 140 °C. Sampling was replicated four times.

### Coupled Gas Chromatography-Electroantennography (GC-EAG)

Female air entrainment extracts were tested for electroantennographic activity on male antennae by coupled GC-EAG using an Agilent 6890 N gas chromatograph equipped with a DB-WAX column (30 m × 0.32 mm i.d.). Helium was the carrier gas and injection was performed in the splitless mode. The column oven temperature program started at 60 °C and increased to 220 °C at 10 °C.min^−1^. The effluent was split between the flame ionization detector (FID) and a heated transfer line to the EAG apparatus. For each test, we co-injected 1 μl aliquots of air entrainment extracts and 10 ng tetradecyl acetate (internal standard) in 1 μl of dichloromethane. For EAG, the male antenna was freshly cut at the base from a live bug, and the tip of the last segment excised to ensure a good connection. The antenna was mounted between two glass capillaries containing Ringer solution. One electrode was grounded, while the other was connected to a high-impedance DC amplifier (IDAC-232, Ockenfels Syntech GmbH, Kirchzarten, Germany). A compound was defined as EAG-active if it evoked an antennal response, distinguishable from background noise, in at least three runs.

### Identification of EAG-Active Compounds

For identification of electrophysiologically active compounds from air entrainment samples, a Hewlett-Packard 5890 series II GC, fitted with a capillary DB-WAX column (30 m × 0.32 mm i.d., 0.5 μm film thickness; J&W Scientific, Folsom, CA) and a cool on-column injector, was coupled to a mass spectrometer (Hewlett-Packard 5972). Ionization was by electron impact at 70 eV. The column oven temperature was maintained at 40 °C for 1 min and then increased at 5 °C.min^−1^ to 250 °C (hold time 17.2 min). The carrier gas was helium. Tentative identification by gas chromatography/mass spectrometry (GC/MS) was confirmed by comparing retention indices of peaks with those of synthetic standards, and by peak enhancement on GC by co-injection with authentic compounds (Pickett 1990) using an Agilent 7890A GC equipped with a cool on-column injector, FID and a 30 m × 0.32 mm i.d., 0.52 μm film thickness DB-WAX column. The oven temperature was maintained at 30 °C for 0.5 min and then programmed at 5 °C.min^−1^ to 150 °C for 0.1 min, then at 10 °C.min^−1^ to 230 °C for 25 min. The carrier gas was hydrogen.

Quantification of compounds was achieved using the multiple-point external standard method, generating calibration curves from synthetic standards.

### Chemicals

Hexyl butyrate, (*E*)-2-hexenyl butyrate, (*E*)-cinnamaldehyde and 1-hexanol (≥96% purity as per the manufacturer) were obtained from Sigma-Aldrich Kft (Budapest, Hungary). (*E*)-4-Oxo-2-hexenal was synthesized as follows. To a solution of 2-ethylfuran (10.00 g, 104.03 mmol) in a mixture of THF (100 ml), acetone (80 ml) and water (40 ml), cooled to −15 °C under nitrogen, was added *N*-bromosuccinimide (27.78 g, 156.04 mmol), followed by pyridine (16.8 mL, 208.06 mmol). The reaction mixture was stirred for 30 mins before being warmed to 0 °C for a further 3 h. The reaction mixture was poured into 0.5 M HCl and extracted with EtOAc. The combined organics were washed with water, dried (MgSO_4_) and concentrated under vacuum. The crude product was purified on silica gel (20% EtOAc in pet ether) to give (*E*)-4-oxo-2-hexenal (4.42 g, 37% yield) as an orange oil.^1^H-NMR (CDCl_3_, 500 MHz): 9.79 (d, 1H, J = 7.2 Hz), 6.90 (d, 1H, J = 16.2 Hz), 6.80 (dd, 1H, J = 16.2 and 7.2 Hz), 2.75 (qu, 2H, J = 7.3 Hz), 1.18 (t, 3H, J = 7.2 Hz)^. 13^C-NMR (CDCl_3_, 500 MHz): 200.38, 193.46, 144.78, 137.30, 34.54 & 7.55. Due to its inherent instability, the compound was stored as a 1:1 solution in dichloromethane at −80 °C until required.

### Field Tests

#### Field Experiment with Live Virgin *A. lineolatus*

This experiment (Experiment 1) was performed at Pusztazámor, Hungary, at the edge of an alfalfa field from July 15 to August 8, 2013. Four different treatments were applied: three virgin females on a green bean pod, three virgin males on a green bean pod, a green bean pod without insects and a blank control. Traps consisted of a plastic roof (27 × 24 cm) with the upper side covered with aluminum foil (I.S.X.-TRADE Kft., Budapest, Hungary) to prevent insolation. On the roof, a transparent sticky PVC sheet (23 × 36 cm) was attached with pegs, its sticky side facing inward. The bugs and pods were placed in 9.5 × 4 cm cylindrical containers made of transparent PVC foil, and closed at both ends with fine mesh. The containers were fixed to the underside of the roof. At each inspection, bean pods and bugs were replaced with fresh ones. One replicate of each treatment was incorporated into a block, within which individual treatments were 5–8 m apart in a randomized arrangement. The distance between blocks was 10–15 m. The experiment was run with 4 blocks. Traps were inspected twice weekly, with insects caught in the sticky insert removed and taken to the laboratory for identification.

#### Field Experiments with Synthetic Compounds

Ternary pheromone baits were prepared as follows: hexyl butyrate, (*E*)-2-hexenyl butyrate and (*E*)-4-oxo-2-hexenal were formulated into 0.7 ml polyethylene vials (No. 730, Kartell Co., Italy) in a 5.4:9.0:1.0 ratio, respectively, and capped. Total load of baits was kept at 50 mg. Binary combinations in Experiment 2 were prepared with the same load of the respective compounds. For Experiment 3, 0.1, 1 or 10 mg of 1-hexanol was added to the ternary pheromone blend in a vial. The dispensers were attached to 8 × 1 cm plastic handles for easy handling when assembling the traps. The dispensers were kept in the shade under the roof of traps and equipped with loosely applied aluminum foil to provide protection from light, since (*E*)-4-oxo-2-hexenal is light-sensitive (Fountain et al. 2014). For Experiment 4, 10 mg of 1-hexanol was added to the ternary pheromone blend either in the pheromone bait or in a separate polyethylene vial. The latter was closed but no shading was added.

(*E*)-Cinnamaldehyde, a known attractant for *A. lineolatus* (Koczor et al. 2012), was also tested as a positive control. Baits were prepared as follows: 100 mg (*E*)-cinnamaldehyde was loaded onto a 1 cm piece of dental roll (Celluron®, Paul Hartmann AG, Heidenheim, Germany), and placed in a polyethylene bag (ca 1.0 × 1.5 cm, 0.02 mm polyethylene foil, (FS471–072, Phoenixplast BT, Pécs, Hungary). Dispensers were heat-sealed and attached to 8 × 1 cm plastic handles for handling when assembling traps. In the field, polyethylene vial dispensers were replaced at intervals of 4–5 weeks and polyethylene bag dispensers replaced every 3–4 weeks. Previous experience has shown that dispensers do not lose attractiveness over this period (Koczor et al. 2012, 2015).

For storage, all dispensers were wrapped singly in pieces of aluminum foil and stored at −18 °C until used. For field testing, CSALOMON® VARL+ funnel traps were used (produced by the Plant Protection Institute, CAR, Budapest, Hungary), which have been proven to be suitable for catching plant bugs (Koczor et al. 2012). A small piece (1 × 1 cm) of household anti-moth strip (Chemotox®, Sara Lee; Temana Intl. Ltd., Slough, UK; active ingredient 15% dichlorvos) was placed in the containers to kill captured insects. The experiments were performed in a randomized complete block design, i.e., one replicate of each treatment was incorporated into a block, with individual treatments 5–8 m apart in a randomized arrangement. Distance between blocks was 10–15 m. To avoid positional effects, trap positions were changed fortnightly. As a rule, traps were inspected weekly, and catches brought to the laboratory, where individuals were sexed and determined to species level.

Details of individual experiments:

- Experiment 2: We tested ternary and binary combinations of hexyl butyrate, (*E*)-2-hexenyl butyrate and (*E*)-4-oxo-2-hexenal (Table 1). Traps were set at the edge of an alfalfa field in the vicinity of Cegléd (Hungary) from 12 July to 24 September, 2018, with 4 blocks.

- Experiment 3: In this experiment, we added 1-hexanol to the ternary pheromone blend, containing hexyl butyrate + (*E*)-2-hexenyl butyrate + (*E*)-4-oxo-2-hexenal. 1-Hexanol was loaded in a 0.1, 1, or 10 mg dose in the same bait dispensers (Table 1). Traps were set at the edge of an alfalfa field in the vicinity of Cegléd (Hungary) from 12 July to 24 September, 2018, with 4 blocks.

- Experiment 4: We added 1-hexanol to the pheromone blend in the same or in separate dispensers to assess if inhibition of *A. lineolatus* catches by 1-hexanol was a result of chemical interactions with pheromone constituents (Table 1). Traps were set at the edge of an alfalfa field in Érd-Elvira major (Hungary) from 15 July to 19 September, 2019, with 5 blocks.

- Experiment 5: In this experiment we tested the effect of the addition of (*E)*-cinnamaldehyde to the ternary pheromone blend (Table 1) on *A. lineolatus* catches. (*E*)-Cinnamaldehyde was added in a separate dispenser. Traps were set at the edge of an alfalfa field in the vicinity of Cegléd (Hungary) from 12 July to 24 September, 2018, with 4 blocks.

- Experiment 6: We tested two doses of (*E*)-4-oxo-2-hexenal in the pheromone blend, as well as whether (*E*)-4-oxo-2-hexenal could be substituted with (*E*)-2-hexenal (Table 1). Traps were set at the edge of an alfalfa field in Érd-Elvira major (Hungary) run from 15 July to 19 September, 2019, with 5 blocks.

**Table 1:** Treatments for Experiments 2–6

treatment/bait composition*	Experiment 2	Experiment 3	Experiment 4	Experiment 5	Experiment 6
HB + E4O2H	+	–	–	–	–
E2HB + E4O2H	+	–	–	–	–
HB + E2HB	+	–	–	–	–
HB + E2HB + E4O2H	+	+	+	+	+
HB + E2HB + E4O2H + 0.1 mg 1-hexanol	–	+	–	–	–
HB + E2HB + E4O2H + 1 mg 1-hexanol	–	+	–	–	–
HB + E2HB + E4O2H + 10 mg 1-hexanol	–	+	+	–	–
HB + E2HB + E4O2H and 10 mg 1-hexanol baits	–	–	+	–	–
(*E*)-cinnamaldehyde	–	–	–	+	–
HB + E2HB + E4O2H and (*E*)-cinnamaldehyde baits	–	–	–	+	–
HB + E2HB + 5× increased dose of E4O2H	–	–	–	–	+
HB + E2HB + (*E*)-2-hexenal	–	–	–	–	+
no bait	+	–	+	+	+

*HB hexyl butyrate, *E2HB* (E)-2-hexenyl butyrate, *E4O2H* (E)-4-oxo-2-hexenal;’+’ indicates the presence of a treatment in an experiment

#### Statistics

Trap catch data were tested for normality by Shapiro-Wilk tests. Since experimental data were not normally distributed, nonparametric tests were used. Inspections with low catches (i.e., accounting for <5% of total catches in an experiment) were excluded from the analysis. Catch data were analyzed by Kruskal-Wallis tests, and differences between treatments were evaluated by pairwise Wilcoxon tests with Benjamini-Hochberg correction. Statistical procedures were conducted using R (R Core Team 2016).

## Results

### Field Experiment with Live Virgin *A. lineolatus*

**Fig. 1:**
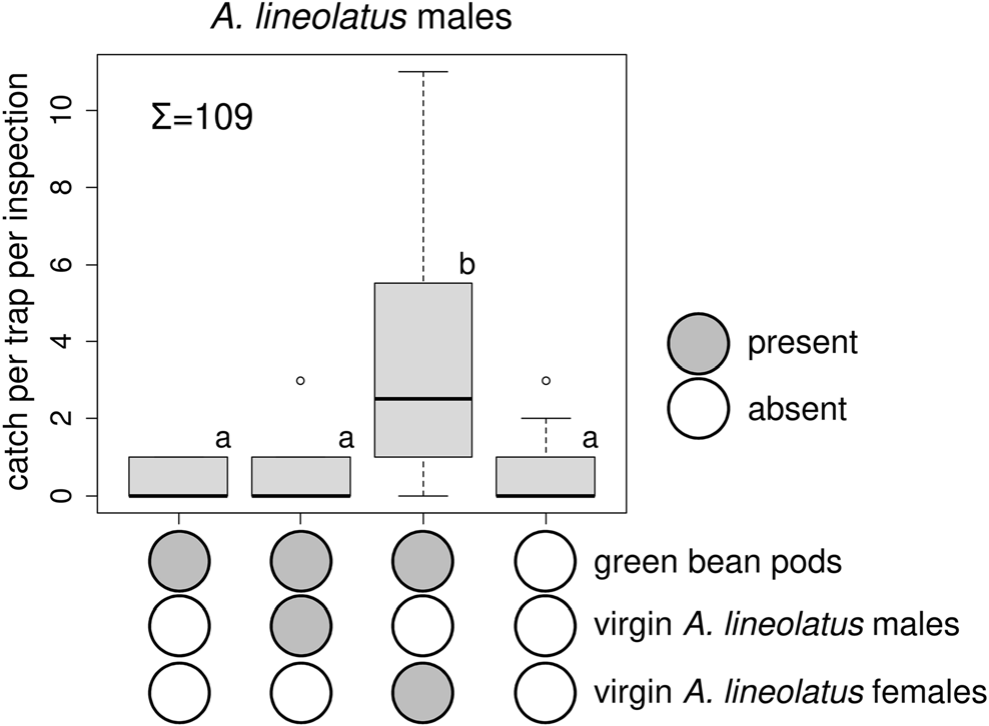
Catches of *Adelphocoris lineolatus* males in traps baited with live virgin *A. lineolatus* males on green bean pods, live virgin *A. lineolatus* females on green bean pods, green bean pods alone or unbaited. Treatments marked with the same letter are not different (Kruskal-Wallis test, pairwise comparison by Wilcoxon test with Benjamini-Hochberg correction at *p* = 0.05) ∑ = total number of *A. lineolatus* males caught in the experiment (box plot diagram indicating median, minimum, maximum, the 1st and 3rd quartiles of catches of the respective treatments)

In Experiment 1, more *A. lineolatus* males were caught in traps baited with live virgin females than in the other treatments; catches in the other treatments did not differ from those in unbaited traps (Fig. 1). No difference was found among treatments for female catch (total female catch: 11, Kruskal-Wallis chi-squared = 4.369, *P* = 0.224, data not shown).

### Analyses of Female Extracts and Identification of EAG-Active Constituents

**Fig. 2:**
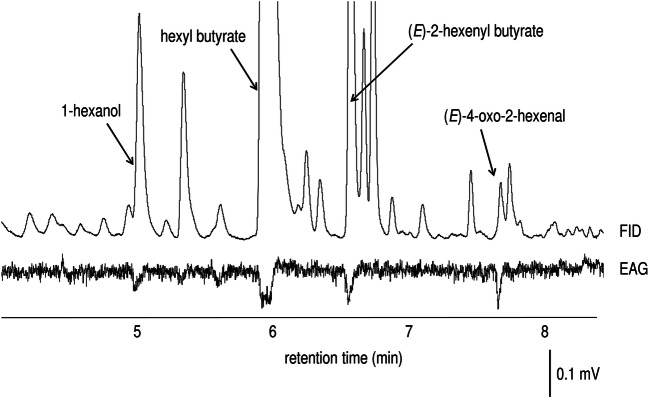
Coupled gas chromatography-electroantennogram (GC-EAG) analysis of a female *Adelphocorus lineolatus* headspace extract, with bioactive peaks labeled. The extract used for GC-EAG shows a ratio of pheromone constituents different from that from air entrainment samples, which were used for quantitative analysis. FID = flame ionization detector

Compounds from female *A. lineolatus* extract that consistently elicited male GC-EAG were identified by GC/MS and GC peak enhancement as hexyl butyrate [Kováts index (KI) on a polar DB-WAX column = 1420], (*E*)-2-hexenyl butyrate (KI = 1478) and (*E*)-4-oxo-2-hexenal (KI = 1592) (Fig. 2). As well as these compounds, another compound elicited stable EAG responses from male antennae and was identified as 1-hexanol (KI = 1360) (Fig. 2). Based on air entrainment samples, the average emission of hexyl butyrate, (*E*)-2-hexenyl butyrate and (*E*)-4-oxo-2-hexenal was 0.27 ± 0.09, 0.45 ± 0.44 and 0.05 ± 0.02 μg/h/female, respectively.

### Field Experiments with Identified Compounds

**Fig. 3:**
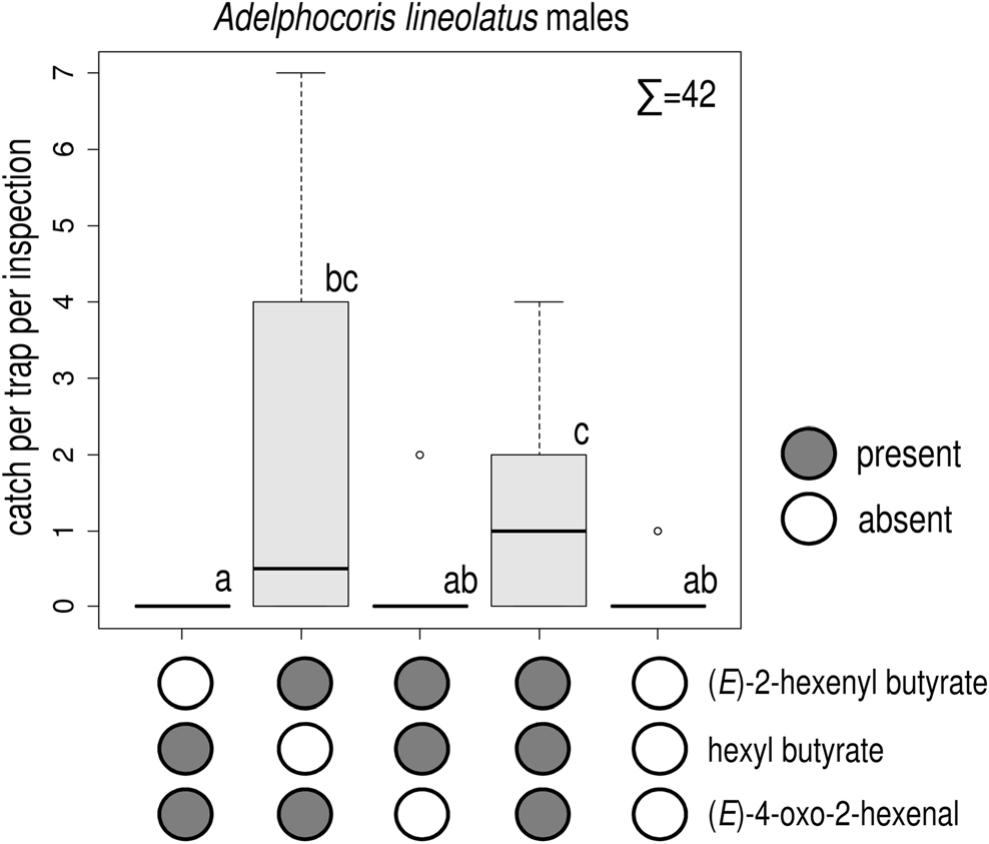
Catches of *Adelphocoris lineolatus* males in traps baited with ternary and binary combinations of hexyl butyrate, (*E*)-2-hexenyl butyrate and (*E*)-4-oxo-2-hexenal and unbaited. Treatments marked with the same letter are not different (Kruskal-Wallis test, pairwise comparison by Wilcoxon test with Benjamini-Hochberg correction at *p* = 0.05) ∑ = total number of *A. lineolatus* males caught in the experiment (box plot diagram indicating median, minimum, maximum, the 1st and 3rd quartiles of catches of the respective treatments)

In Experiment 2, the ternary blend caught more *A. lineolatus* males than did unbaited traps (Fig. 3). Catches by the binary combination of (*E*)-2-hexenyl butyrate and (*E*)-4-oxo-2-hexenal were lower but did not differ from those by the ternary blend. Only few females were caught, with treatments not differing from each other (total female catch: 15, Kruskal-Wallis chi-squared = 4.077, *P* = 0.396, data not shown).

**Fig. 4:**
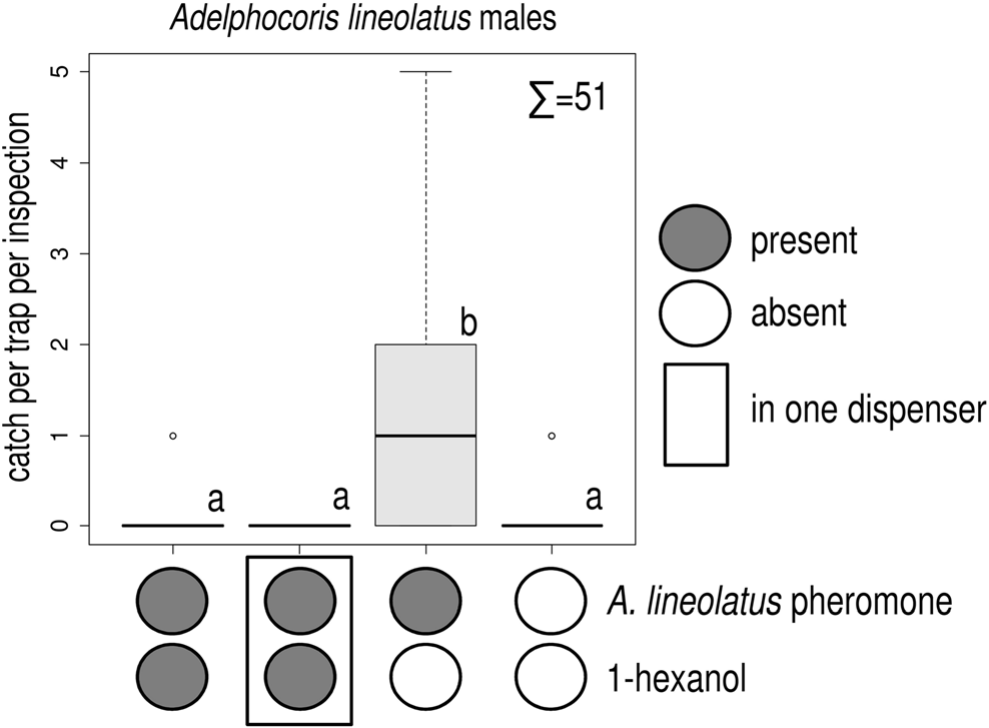
Catches of *Adelphocoris lineolatus* males in traps baited with a ternary pheromone blend, with addition of 1-hexanol, and unbaited. The 1-hexanol was added either in the same or a separate dispenser. Treatments marked with the same letter are not different (Kruskal-Wallis test, pairwise comparison by Wilcoxon test with Benjamini-Hochberg correction at *p* = 0.05) ∑ = total number of *A. lineolatus* males caught in the experiment (box plot diagram indicating median, minimum, maximum, the 1st and 3rd quartiles of catches of the respective treatments)

In Experiment 3, addition of 1 or 10 mg of 1-hexanol to the ternary blend decreased male catch (Table 2). For female catch, no difference was found among treatments. In Experiment 4, addition of 1-hexanol to the ternary pheromone blend reduced catches of *A. lineolatus* males, regardless of whether the compound was loaded into the same or a separate dispenser. Only treatments containing the ternary pheromone blend alone caught more *A. lineolatus* males than unbaited traps (Fig. 4). For females, treatments did not differ (total female catches: 6, Kruskal-Wallis chi-squared = 2.106, *P* = 0.551, data not shown).

**Table 2:** Catches of *Adelphocoris lineolatus* males and females in traps baited with a ternary pheromone blend and different doses of 1-hexanol (total catch = 45 *A. lineolatus*)

		catch per trap per inspection ± SE*	
		*Adelphocoris lineolatus*	
pheromone blend	dose of 1-hexanol	males	females
present	–	1.25 ± 0.43 b	0.62 ± 0.32 a
present	0.1 mg	0.75 ± 0.37 b	0 ± 0 a
present	1 mg	0 ± 0 a	0 ± 0 a
present	10 mg	0 ± 0 a	0 ± 0 a

*Treatments marked with the same letter are not different (Kruskal-Wallis test, pairwise Wilcoxon test with Benjamini-Hochberg correction at *P* = 0.05)

**Fig. 5:**
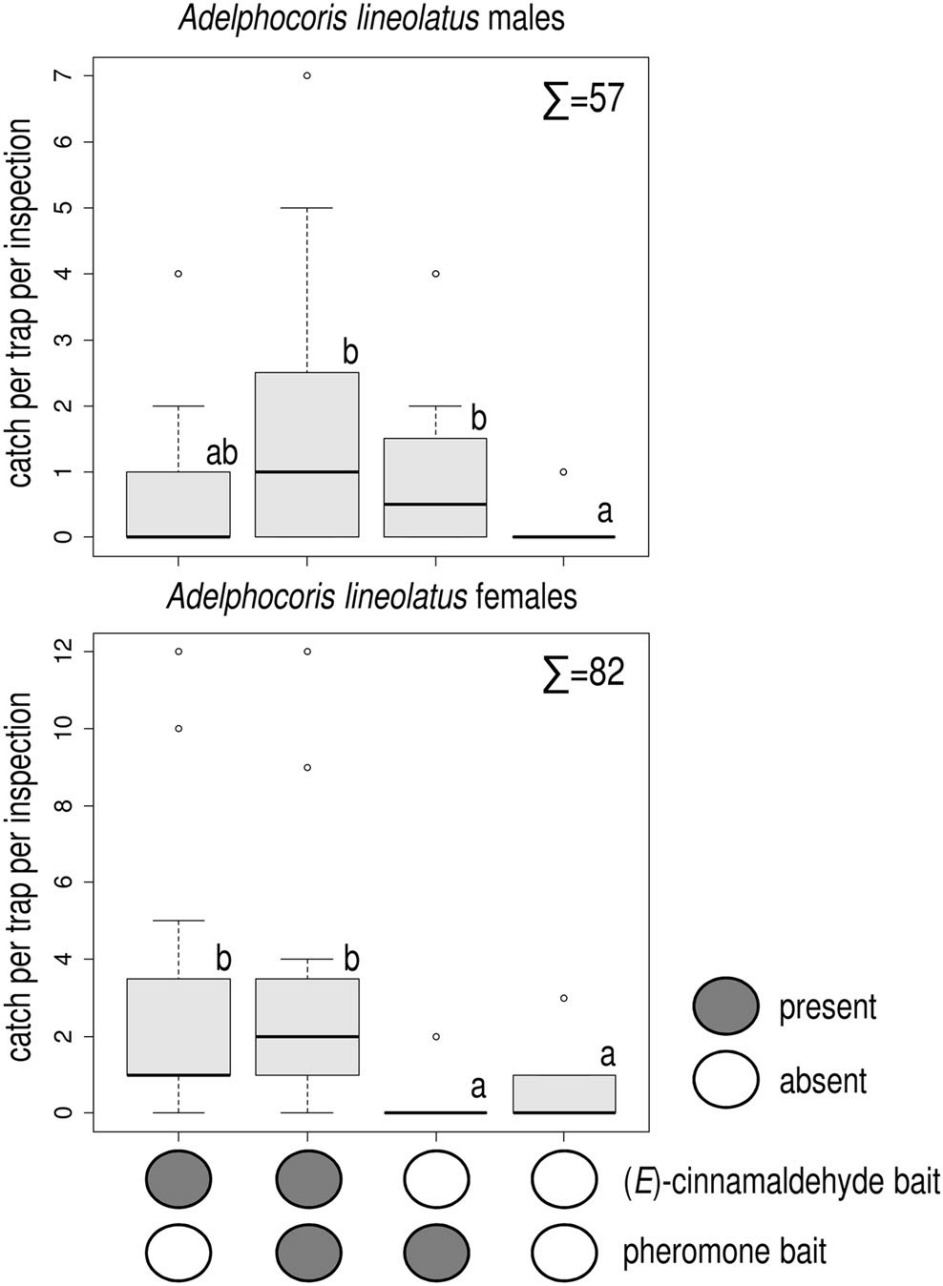
Catches of *Adelphocoris lineolatus* males and females in traps baited with a ternary pheromone blend, (*E*)-cinnamaldehyde, their combination and unbaited. Treatments marked with the same letter are not different (Kruskal-Wallis test, pairwise comparison by Wilcoxon test with Benjamini-Hochberg correction at p = 0.05) ∑ = total number of *A. lineolatus* males/females caught in the experiment (box plot diagram indicating median, minimum, maximum, the 1st and 3rd quartiles of catches of the respective treatments)

In Experiment 5, treatments containing the ternary blend caught more *A. lineolatus* males than did unbaited traps (Fig. 5). Addition of (*E*)-cinnamaldehyde to the ternary blend gave no increase in male catch compared to the ternary blend alone. For females, treatments containing (*E*)-cinnamaldehyde caught more individuals than unbaited traps, irrespective of the presence or absence of pheromone baits.

**Fig. 6:**
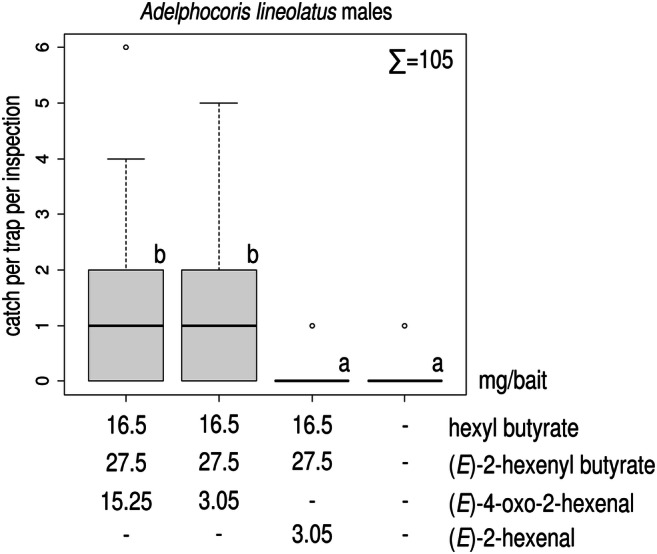
Catches of *Adelphocoris lineolatus* males in traps baited with a ternary pheromone blend with a standard dose of (*E*)-4-oxo-2-hexenal, with a 5-fold increased dose of (*E*)-4-oxo-2-hexenal, with (*E*)-2-hexenal substituted for (*E*)-4-oxo-2-hexenal and unbaited. Treatments marked with the same letter are not different (Kruskal-Wallis test, pairwise comparison by Wilcoxon test with Benjamini-Hochberg correction at p = 0.05) ∑ = total number of *A. lineolatus* males caught in the experiment (box plot diagram indicating median, minimum, maximum, the 1st and 3rd quartiles of catches of the respective treatments)

In Experiment 6, treatments containing the ternary blend caught more *A. lineolatus* males than did the unbaited traps. Baits with the higher amount of (*E*)-4-oxo-2-hexenal did not catch more *A. lineolatus* males than those with the lower dosage (i.e., the original ternary blend; Fig. 6). Catches of traps baited with the blend substituting (*E*)-2-hexenal for (*E*)-4-oxo-2-hexenal caught similar numbers of males as did the unbaited traps (Fig. 6). For females, no difference was found among treatments (total female catch: 5, Kruskal-Wallis chi-squared = 2.038, *P* = 0.564, data not shown).

## Discussion

Our results on central European populations of *A. lineolatus* confirm the identity of hexyl butyrate, (*E*)-2-hexenyl butyrate and (*E*)-4-oxo-2-hexenal as female-produced pheromone components of *A. lineolatus,* as reported previously from east Asian populations (Zhang et al. 2015a). The relative importance of the compounds identified was also similar in the present study, as binary blends from which either (*E*)-2-hexenyl butyrate or (*E*)-4-oxo-2-hexenal were missing were not active, whereas binary combination of these compounds and the ternary blend were. Thus, it appears that populations from central Europe and east Asia are similar with respect to the chemistry of their pheromonal communication.

The above three compounds are sex pheromone components of several other plant bug species, including *L. rugulipennis* (Innocenzi et al. 2005) and *L. pratensis* (Linnaeus) (Fountain et al. 2014), which may occur in the same habitats as *A. lineolatus*. Fountain et al. (2014) reported that the ratios of the same three sex pheromone components for the closely related *Lygus*, *Lygocoris* and *Liocoris* species, were less important. Thus, it is likely that other means of communication may also be important in mate recognition in *A. lineolatus*, as found for *Lygocoris pabulinus* (Linnaeus) (Drijfhout and Groot 2001) and *L. rugulipennis* (Koczor and Cokl 2014).

Based on the findings of Yasuda and Higuchi (2012) on *S. rubrovittatus*, we tested a higher dosage of (*E*)-4-oxo-2-hexenal in the pheromone blend; however, this did not result in catches of more males. Since a compound may have multiple functions and (*E*)-4-oxo-2-hexenal is thought to be important in defense (Moreira and Millar 2005), we tested blends in which (*E*)-4-oxo-2-hexenal was substituted by (*E*)-2-hexenal, a more stable compound: the substituted blend did not attract males.

1-Hexanol was also found in air entrainment samples of female *A. lineolatus* and elicited EAG responses from male antennae. Surprisingly, when the compound was tested in combination with the ternary pheromone blend, it resulted in a decrease in male catch. Subsequent field experiments, in which the compound was tested in the same or separate dispensers showed that this was likely due to a biological response and not to a chemical reaction in the lure. However, we cannot rule out that the compounds might react in the air. In their laboratory study on host plant volatiles, Sun et al. (2013) found that more *A. lineolatus* adults chose a solvent control over 1-hexanol in Y-tube olfactometer tests, indicating a repellent-like effect, supporting our finding.

The ecological role of 1-hexanol for *A. lineolatus* is uncertain. Host plant volatiles are known to affect sex pheromone production and activity in insects (Landolt and Phillips 1997); for instance, in *L. rugulipennis* a closely related plant bug species, Frati et al. (2009) found that host plant odors evoked increased sex pheromone production in females. Thus, it is possible that a compound indicating unfavorable conditions of a host may negatively affect attraction of males to sex pheromone. Another potential explanation is that the antagonistic effect could have been functionally important in the past. For instance, if an ancestor of *A. lineolatus* was using 1-hexanol as a pheromone component, the compound could have become antagonistic during speciation as indicative of the ancestral species. Interestingly, 1-hexanol was found in gland extracts of a closely related eastern Asian species, *A. suturalis* (Zhang et al. 2014). Nevertheless, as air entrainment extracts in this study were prepared from live bugs on green bean pods, the compound may also be connected to other activities, such as feeding.

Several reports have demonstrated the synergistic effect of plant volatiles on insect attraction to sex pheromones (Landolt and Phillips 1997). This, however, was not the case for *A. lineolatus* males as addition of the known floral attractant, (*E*)-cinnamaldehyde, to the pheromone had no significant effect on catch. On the other hand, the presence of the sex pheromone did not affect catch of females to (*E*)-cinnamaldehyde. This lack of interaction between the chemicals may open up opportunities for monitoring both sexes using a combination of sex pheromone and (*E*)-cinnamaldehyde.

Pheromones are important in monitoring or direct control (e.g., mating disruption) of insect pests (Witzgall et al. 2010). Whereas monitoring of plant bugs may prove to be an important tool in agriculture, mating disruption may not be economically feasible, as suggested by Yasuda and Higuchi (2012). In the case of *A. lineolatus*, one problem could be the instability of (*E*)-4-oxo-2-hexenal affecting its storage and bait longevity. A further problem for health and safety could be the irritating property of this compound. Substitution of this compound with a more stable alternative may be a solution; however, as our study has shown, more work is needed to screen for feasible substitutes. Finally, 1-hexanol may be suitable for use as a sex pheromone antagonist in, for example, mating disruption. Experiments are underway to assess its potential.

## Supplementary Information

ESM 1 (PDF 115 kb)

## Data Availability

Not applicable.
