# Supplementary material for: Sex Pheromone of the Alfalfa Plant Bug, Adelphocoris lineolatus: Pheromone Composition and Antagonistic Effect of 1-Hexanol (Hemiptera: Miridae)
Source: J Chem Ecol. 2021 Apr 19;47(6):525–33. doi: 10.1007/s10886-021-01273-y (PMC8217015; doi:10.1007/s10886-021-01273-y)
Supplement: Supplementary file 1 — (PDF 115 kb) [file 10886_2021_1273_MOESM1_ESM.pdf]

Electronic Supplementary Material

Title: Sex pheromone of the alfalfa plant bug, *Adelphocoris lineolatus*: pheromone composition and antagonistic effect of 1-hexanol (Hemiptera: Miridae)

Journal: Journal of Chemical Ecology

Authors: Sándor Koczor*[1], József Vuts[2], John C. Caulfield[2], David M. Withall[2], André Sarria[2, 3], John A. Pickett[2, 4], Michael A. Birkett[2], Éva Bálintné Csonka[1], Miklós Tóth[1]

[1]Plant Protection Institute, Centre for Agricultural Research, H-1022 Herman Ottó u. 15, Budapest, Hungary; *corresponding author: koczor.sandor@atk.hu

[2] Department of Biointeractions and Crop Protection, Rothamsted Research, Harpenden, Hertfordshire AL5 2JQ, UK

[3] Present address: Biobab R&D, S.L., Calle Patones, s/n. Parcela 28.3 PI Ventorro del Cano, 28925 Alcorcón, Madrid, Spain

[4] Present address, School of Chemistry, Cardiff University, Cardiff, CF10 3AT, Wales, UK

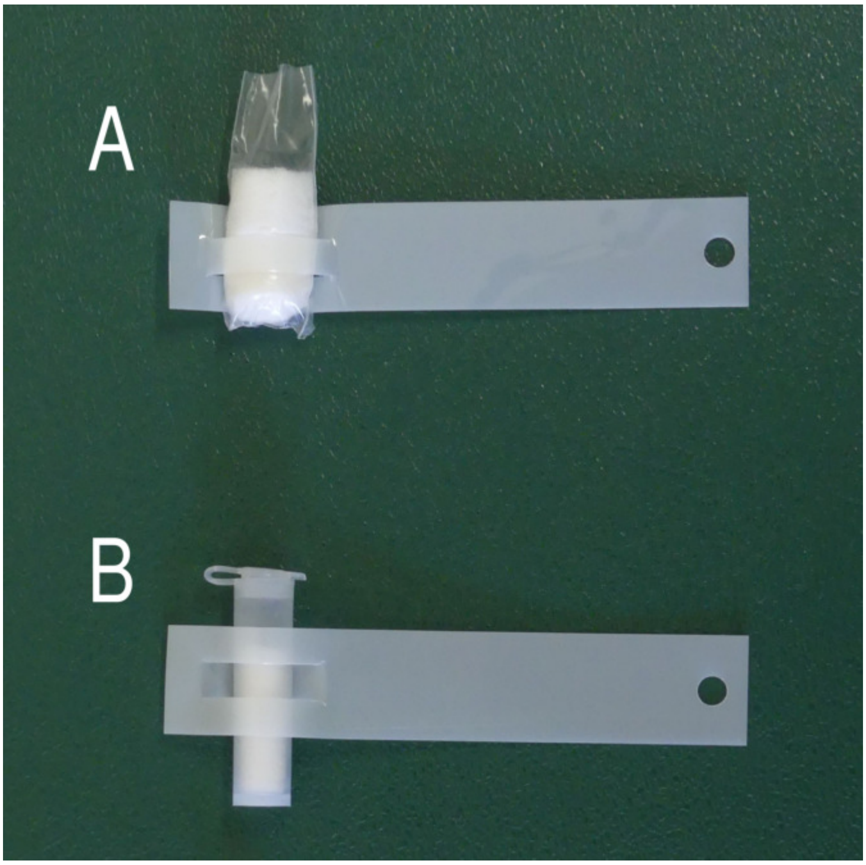

Supplementary Fig. 1: Dispenser types applied in the study. A: polyethylene bag, B: polyethylene vial
